# Resolving Strategic Dilemmas in Ambidextrous Organizations: An Integrated Second-Order Factor Model Perspective

**DOI:** 10.3389/fpsyg.2022.797645

**Published:** 2022-03-10

**Authors:** Rongning Cao, Ruchuan Jiang

**Affiliations:** ^1^School of Business, Jiangsu University of Technology, Changzhou, China; ^2^Business School, China University of Political Science and Law, Beijing, China

**Keywords:** ambidextrous organization, strategic dilemma, integrated mechanism, second-order factor model, performance measurement balance, innovation ambidexterity

## Abstract

Drawing on relevant literature, this study investigates the process of realizing innovation ambidexterity (IA) by proposing a theoretical model and adopting a specifically integrated mechanism with the aim to resolve strategic dilemmas in ambidextrous organizations (AOs). We analyzed a sample of 136 cross-sectional surveys collected from business managers of 132 medium- and high-tech firms in China by employing a structural equation model combined with moderation analysis to test our hypotheses. Our findings indicate that the second-order theoretical model fits the data well and AO, represented by a higher-order construct, positively affects IA. Instead of structural ambidexterity, balanced contextual ambidexterity and radical performance management can be effectively applied as the factors of the second-order construct; the design comprising balanced contextual ambidexterity and performance management is thus helpful in resolving strategic dilemmas. Our findings demonstrate that Chinese firms, as technology latecomers, are more inclined to conduct near-radical innovation. The risk of exploration crowding out exploitation efforts exists in Chinese high-tech firms. Furthermore, we provides greater insights into the moderating impact of intra-organizational practice on IA based on the fact that performance measurement balance (PMB) did not directly influence the achievement of IA and clarifies the positive role that PMB plays in improving IA.

## Introduction

This study pertains to ambidexterity in organizational innovation. As for the notion of ambidexterity, it refers to the integration and reconciliation of both exploitative and exploratory activities, which can produce incremental and radical innovations, respectively (Smith and Tushman, 2005; Jansen et al., 2012). Organizational ambidexterity refers to an organization’s capacity to address equally contradictory objectives (Gibson and Birkinshaw, 2004; Kauppila, 2010; Birkinshaw and Gupta, 2013). In view of the importance of organizational ambidexterity, many researchers have sought to determine how a firm could realize organizational ambidexterity and achieve innovation outcomes. According to Bedford et al. (2016), innovation ambidexterity is achieved incremental and radical innovation outcomes. However, the achievement of IA may involve multiple objectives, including contradictory ones. March (1991) insists that firms face the strategic dilemma of binary decision-making because exploitative and exploratory activities compete for scarce resources. The dilemma of balancing such activities is considered as one of the toughest managerial challenges in sustaining a firm’s competitive advantage (Williamson, 1999). Prior dilemmas research indicates that conflict associated with ambidextrous innovation activity is higher in organizations that engage in simultaneous exploration and exploitation (Smith, 2014; Bedford et al., 2016). Recent studies also point out that ambidextrous innovative practices are particularly challenging for small and medium-sized enterprises due to their serious financial constraints and information asymmetries (Barbaroux, 2014).

How firms achieve superior performance remains at the heart of strategic management (Schilke et al., 2018). To address this strategic dilemma, He and Wong (2004) highlight how competing for firms’ scarce resources results in the need to manage the trade-offs between exploitation and exploration. Other researchers have also proposed that exploitation and exploration could be balanced by creating a behavioral context that is a combination of discipline, stretch, support, and trust (Gibson and Birkinshaw, 2004). Besides studies mentioned above, similar research has also been done recently. Despite insightful contributions made by related research, the previous research still offers a partial picture as to how to resolve strategic dilemmas in ambidextrous organizations.

First of all, these studies do not explicitly focus on a configurational approach accounting for the non-linear complex interplay between constitutive elements. Then, previous studies have produced contradictory results about a firm’s superior performance (Helfat et al., 2007). Some researchers attribute this problem to the fact that most studies have been dominated by theoretical discussions, and relatively weak empirical support (Protogerou et al., 2011). In fact, according to the findings of Fiss (2011), causal asymmetry may occur as an explanation to organizational outcomes. And third, how an integrated mechanism is beneficial for resolving organizational strategic dilemmas remains unclear because only a few studies have comprehensively investigated the associations among social support (SS) of competence ambidexterity (the simultaneous pursuit of exploitation and exploration activity), performance management (PM), and IA (Agostini et al., 2016; Bedford et al., 2016). Only a few studies have provided evidence—albeit limited—that enhances our understanding of which structures and processes allow firms to resolve strategic dilemmas arising from the tension between exploitation and exploration (O’Reilly and Tushman, 2013). So, the long-ignored questions emerge: whether an integrated competence ambidexterity and performance management can lead to IA? Whether a balanced performance measurement is beneficial? There is no previous research on how an integrated mechanism of innovation management influences IA and how to manage the ambidextrous organization (AO) of medium- and high-tech firms in a non-Western and emerging economy. Contrary to the integration of innovative business models (Vendrell-Herrero et al., 2018), we intend to integrate management practices. Hence, this study focuses on the above literature gaps, detailing how to manage the tension of exploitation and exploration in China by focusing on organizational feature and its incentive effect.

China has successfully transformed itself from imitator to innovator (Mathews, 2006). An increasing number of leading Chinese firms have progressed from simply imitating others to innovating alongside them. Some of these firms have even become global innovation leaders (Guo and Zheng, 2019). Hence, the research findings that are relevant to Chinese firms can provide theoretical and practical implications regarding the innovation management of AO in the context of a non-Western technology latecomer. Examining the innovation management of Chinese enterprises is of great significance potentially for not only enterprises in other emerging economies but also companies in developed countries.

This study attempts to explain how to resolve organizational strategic dilemmas in an integrated way while addressing research gaps in the current ambidextrous innovation management literature by placing it in a broader behavioral context. Ajzen (1991) develops the theory of reasoned action and the theory of planned behavior to predict and explain human intention and behavior. Generally, related theories posit that individuals’ behaviors are depended on their attitudes, which evaluate their tendency toward behavior. In fact, behavioral theory is particularly well positioned to help researchers understand how management accounting practices are developed in response to changes in the organizational context (Hall, 2016). To achieve the research objective, we collected 136 cross-sectional survey observations from managers of 132 Chinese medium- and high-tech firms and tested them using a balanced contextual approach. A longitudinal study finds that during the transition phase of a business model, the firm involved both incremental evolution and radical transformation of products (Spieth et al., 2014). Innovation ambidexterity is particularly associated with medium- and high-tech industries where incremental improvements are important and the firms compete in new technologies where radical innovations are also needed at the same time (Kaulio et al., 2017).

This study makes following contributions. First, we challenge the long-held viewpoint by revealing the risk of exploration crowding out exploitation efforts. We find that RPM, rather than IPM, plays a more active role in the integration of management mechanism. Although some researchers argue that contextual ambidexterity (the use of behavioral and social means to integrate the disparate demands of different strategies) emerges when managers shape an organizational context with proper degrees of SS and PM (Ghoshal and Bartlett, 1994; Gibson and Birkinshaw, 2004), few extant papers have provided further insights into the mechanism by which the trade-offs between exploitation and exploration is realized or managed and empirically explained the effect of adopting a specifically integrated mechanism on IA. After all, the importance of adopting a balanced and comprehensive set of ambidextrous organizational approaches simultaneously has been emphasized in literature (Kauppila, 2010). Meanwhile, the existing research upholds that the tendency toward the crowding out of exploration exists due to short-term certainty of exploitation success and uncertain payoffs of exploration. However, we come to a different conclusion.

Second, although the extant literature has demonstrated a positive relationship between AO and IA (McCarthy and Gordon, 2011; Wang and Rafiq, 2014), it has failed to conceptualize AO as a second-order construct reflected through a specifically balanced and contextual approach in an integrated structural equation model (SEM). Contextual ambidexterity is regarded as a higher-order approach, which may generate lasting benefits in achieving organizational effectiveness (Birkinshaw and Gibson, 2004). However, questions regarding what specific context is essential to dealing with the tension remain unclear (Yang et al., 2015). This study explores the question of whether it might bring into tension incentives to simultaneously pursue exploitation and exploration and manage the exploitation/exploration dilemma, responding to emerging calls for more empirical studies on the relationship between AO and IA (Mei et al., 2013). Our findings reveal the importance of organizational feature (i.e., AOs) in the process of realizing IA, which means that in order to align exploitation and exploration activity, it is necessary to provide incentive to tailor employee’s behavior to organizational management objective.

Third, this paper further investigates the moderating role of PMB and analyses its indirect effect on the achievement of IA. Performance measurement balance (PMB) provide a balanced representation of the organizational efforts toward incremental and radical innovation, which may be expected to play a moderating role in the achievement of ambidextrous innovation outcome due to its probable motivational impact on employee’s behavior. Prior studies (O’Reilly and Tushman, 2008; Wang and Rafiq, 2014) only take contextual variables as first-order factors and analyze the direct causal relationship between AO and IA in a higher-order model. However, this paper provides greater insights into the moderating impact of intra-organizational practice on IA based on the fact that PMB did not directly influence the achievement of IA and clarifies the role that PMB plays in improving IA.

The remainder of this paper is organized as follows. Section “Literature Review and Hypotheses Development” reviews the relevant theories and prior studies and develops hypotheses. Section “Research Method and Design” introduces the research design and data analysis method. Section “Results” presents the results. Section “Discussion” discusses the significant findings and implications.

## Literature Review and Hypotheses Development

### The Definition of an Ambidextrous Organization

Lin and McDonough (2011) indicate that ambidexterity could be defined as behavioral, structural, and performance implications of being ambidextrous that may lead to the resolution of tensions between exploitation and exploration activities. More specifically, Eisenhardt and Martin (2000) suggest that overall capabilities require a blend of exploitation and exploration. Motivation is also believed to be important for individuals to engage in behaviors, for example, by using some type of social mechanism (Lawson et al., 2009).

Gibson and Birkinshaw (2004) propose that it is possible to balance the pursuit of exploitation and exploration by creating a behavioral context. Additionally, Atuahene-Gima (2005) points out that the key to resolving innovation dilemma may be organizational factors that can ensure simultaneous investment in both the exploitation of innovation capabilities in existing products and the exploration of new ones. In general, there is a growing literature to support the need to balance exploitation with exploration.

However, empirical evidence for ambidexterity issues has, so far, been largely anecdotal and inconclusive (He and Wong, 2004). The issue of how to manage the contradictory tensions between exploitation and exploration effectively, and further convert these opposing objectives into realized IA outcomes, remains relatively unexamined (Smith, 2014).

Hence, based on a comprehensive view of ambidexterity, this study focuses on independent legal organizations and adopts the term “ambidextrous organizations” (AOs), which refers to behavioral and/or contextual ambidexterity, and focuses on the organizational feature leading to the resolution of tensions to realize multiple albeit contradictory objectives (Lin and McDonough, 2011; Chang and Hughes, 2012; Agostini et al., 2016).

### Ambidextrous Organization as a Higher-Order Construct

According to prior studies (McCarthy and Gordon, 2011), SS refers to the extent to which management devotes considerable effort in both developing subordinates and innovation activities. PM involves a context where management uses corporate objects and performance indicators to run the business. As Kauppila (2010) emphasizes, it is important for an AO to adopt a comprehensive set of organizational approaches containing both SS and PM. In this respect, O’Reilly and Tushman (2013) also point out that an organization needs to combine both contextual and structural solutions, instead of single configurations alone, to pursue ambidexterity. Similarly, Ortiz de Guinea and Raymond (2020) combine the two dimensions of IT ambidexterity (i.e., IT capabilities for exploitation and IT capabilities for exploration) with other strategic constructs to form a configurational approach.

From a higher-order conceptualization perspective, Koufteros et al. (2009) illustrate the efficacy of higher-order modeling for handling important conceptual and methodological issues relating to uni-dimensionality, discriminant, convergent, and multicollinearity concerns. As explained by Gerbing et al. (1994), variables satisfying rigorous statistical criteria and exhibiting superb psychometric properties can be concocted as the constituent facets of second-order constructs. To satisfy multiple-indicator uni-dimensionality concerns, researchers usually integrate variables to correspond to a substantive construct of interest in evaluating higher-order conceptualization. Other studies focusing on contextual ambidexterity have been also operationalized as a second order construct with two sub-dimensions—exploitation and exploration (e.g., Soto-Acosta et al., 2018).

Although the aforementioned themes are frequently encountered in studies, few papers on ambidexterity refer to AO explicitly for illustrating context used in a higher-order model structure, let alone constructs of a balance and comprehensive context. Only Agostini et al. (2016) investigate whether the ambidextrous organization can be meaningfully described by a higher-order structure comprising structural and contextual solutions. In fact, according to McCarthy and Gordon (2011), the AO construct should be a higher-order construct characterized by a combination of SS and PM. O’Reilly and Tushman (2008) also argue that the resolution of tensions between exploitation and exploration requires organizations to be integrated around a culture associated with the complementary context. In addition, Birkinshaw and Gupta (2013) call for studies to examine ambidexterity as a multi-level construct characterized by the adoption of different organizational solutions for dealing with the tensions. However, the literature on ambidexterity neglects higher-order modeling and empirical research has deviated from adopting a combination of organizational structural and contextual practices, treating them as single different approaches without explicit conceptual advance (Agostini et al., 2016).

Psychology theories have been used to study management accounting practices (Birnberg et al., 2007), with some studies informing the understanding of how and why such practices influence individual behavior. Basing on goal setting theory, Webb (2004) finds that the perceived strength of the cause-effect link between non-financial and financial measures in a strategic performance measurement system affects individuals’ commitment to goals. Similarly, Hall (2008) finds that a comprehensive performance measurement system is positively associated with process clarity. Meanwhile, motivational theory (e.g., Hebda et al., 2012; Alexander and Van Knippenberg, 2014), which suggests that innovators are driven by their strong motivations arising from intense curiosity, determination, and passion for their work, may provide a theoretical explanation for why a certain combination of management accounting contexts affects organizational performance *via* its influence on the actions of individuals (Hall, 2016).

In line with the emphasis of previous literature on a comprehensive integration of organizational approaches and their effects on individual behavior, we suggest that it is beneficial for organizations to execute management accounting practices more comprehensively because it not only generates tension incentives to simultaneously pursue exploitation and exploration, but also influences individual behavior in AO. According to Bedford et al. (2019), firms emphasizing competence ambidexterity provide a balanced representation of the organizational efforts and the balance between measures (including performance measures incentivizing both innovations and innovation ambidexterity measures) is also an important consideration for ambidextrous firms. Wang and Rafiq (2014) use competence exploration and competence exploitation as two aspects of contextual ambidexterity to explore the relationship between SS of competence ambidexterity and innovation outcome. In this study, we propose that AO can be integrated as a second-order construct in terms of a balanced and comprehensive context. This construct consists of SS and PM. Given the importance of measurement diversity, we measure SS through two dimensions—IIS and RIS. PM is also measured using two dimensions—incremental performance measurement (IPM) and radical performance measurement (RPM). Hence, the following hypothesis is proposed:

H1: An ambidextrous organization (AO) is a higher-order specification including first-order factors consisting of social support (SS) and performance management (PM).

### Ambidextrous Organizations and Innovation Ambidexterity

The relationship between the pursuit of ambidexterity and innovation performance has been widely researched in prior studies. With respect to types of ambidexterity (i.e., either simultaneous or sequential or both), there are opposing views in the strategic management literature regarding which demands organizations need to focus on in order to increase their performance (Ortiz de Guinea and Raymond, 2020). One stream of the literature states that organizations, if ambidextrous, are capable of pursuing simultaneous ambidexterity. As suggested by March (1991), the exploitation and exploration may cause different effects on the firm’s performance. Similarly, Leonard-Barton (1992) suggests that the right blend of innovation activities could achieve innovation outcomes. Junni et al. (2013) further argue that resolving contrasting tensions has generally been demonstrated to contribute to superior ambidexterity performance.

Dynamic capability (DC) is defined as the firm’s ability to integrate internal and external organizational competences to address rapidly changing environments (Teece et al., 1997). The integration can be regarded as a DC process (Eisenhardt and Martin, 2000). From this perspective, drawing a general overview of the quantitative evidence on the DC–performance relationship through a synthesized lens seems pertinent in order to reveal the current concerns and gaps (Baía and Ferreira, 2019). Firms should also align exploitation and exploration capabilities to achieve complementary effects (Smith and Lewis, 2011).

Empirical research into these competing views of ambidexterity has yielded inconsistent and conflicting results for the most part (Ortiz de Guinea and Raymond, 2020). And, despite these inconsistent and conflicting results, studies of the achieved innovation outcomes from pursuing ambidexterity have been varied. Simsek et al. (2009) find that simultaneously combining exploitation and exploration can improve the satisfaction of customers. Schulze et al. (2008) propose that ambidexterity has a positive effect on subjective ratings of performance measured as a latent composite of operational and strategic planning. Further, the combined effect of these practices enables firms pursuing exploitation and exploration simultaneously to obtain different types of innovations at the same time (Gupta et al., 2006). Cao et al. (2009) adapt business performance comprising financial and non-financial items to measure firm performance. From this perspective, the findings by Lin et al. (2013) show that the positive relationship between organizational ambidexterity and performance exists and is strengthened when ambidexterity is found at different organizational levels. Some researchers find that contextual ambidexterity is of importance for new product innovation in high-tech firms and mediates the relationship between ambidextrous organizational culture and new product innovation outcomes (Wang and Rafiq, 2014). Additionally, contextual ambidexterity is regarded as a higher-order approach that may generate lasting benefits of organizational effectiveness (Birkinshaw and Gibson, 2004). As to contextual aspects affecting manufacturing multinational enterprises (MMNEs), Bustinza et al. (2019) point out that contextual factors affecting the development of innovation outcomes are related to the different strategic objectives of MMNEs.

Some studies also draw on the motivation theory of psychology to formulate hypotheses and generate expectations. For example, Perera et al. (1997) draw on the motivational process and develop the expectation that the increasing use of non-financial performance measurements is associated with enhanced performance because such measurements are important in generating and directing managerial actions toward the attainment of strategic priorities. Chen et al. (2020b) find that alignment coping combination enhances IA by reshaping an entrepreneur’s cognitive structure. Also, Pryor et al. (2021) explain how organizational ambidexterity can be affected by top decision makers’ motivations. Likewise, psychology theory is employed (implicitly or explicitly) in contingency-based management accounting research to examine organizational performance as the dependent variable (Gerdin and Greve, 2004).

In view of the reactions to the potential conflicts between different types of accounts, Hall (2016) points out that different accounts can generate cognitive conflict, and managing conflict between accounts is particularly important because established management accounting practices play a role in influencing organizational outcomes. The competing views and conflicting results regarding ambidexterity has led to calls for research adopting a configurational approach and a shift toward an asymmetric understanding of how the different types of ambidexterity relate to performance outcomes could resolve prior conflicting results (Fiss, 2011). Overall, it is feasible to explain the causal connection between comprehensive management accounting practices and performance, considering simultaneous ambidexterity theory and psychology theory. Hence, the following hypothesis is proposed:

H2: An ambidextrous organization (AO) is positively associated with innovation ambidexterity (IA).

### The Moderating Role of Performance Measurement Balance

As suggested by Gibson and Birkinshaw (2004), both exploitation and exploration are considered important activities for improving business performance and sustaining competitive advantage. Their simultaneous pursuit often leads to contradictions and inconsistencies owing to competition for scarce resources (Simsek et al., 2009). In order to handle these competing claims, firms need to find the right combination of different types of practice.

Ambidextrous organization, as a whole, is a higher-order specification integrated with SS and PM that directly influences IA. To some extent, it is beneficial to integrate different types of practice to resolve strategic dilemmas in AOs. However, tension between exploitation and exploration may still occur because an excessive focus on exploitation may result in competency gaps; moreover, the tension may result in a trap, as noted by Levitt and March (1988), when firms simultaneously engage in these two innovation activities.

After reviewing previous research on management control systems (MCSs) and performance measurement systems (PMSs), we find that the design or use of PMS influences organizational innovation outcomes to a large extent. Earlier research focuses on how firms address the performance measurement of higher diversity. Said et al. (2003) point out that firms employing a combination of financial and non-financial performance measurements have significantly higher mean levels of returns on assets, which means the broader set of performance measurements is beneficial for improving organizational performance. Lillis and van Veen-Dirks (2008) reveal that PMSs with a higher diversity of broad-scope metrics exist in firms emphasizing multiple strategic priorities.

However, it may be problematic for firms pursuing ambidexterity to design a PMS with greater diversity of performance measurements (Bedford et al., 2016) because March (1991) indicates that uncertainty of exploration benefit could enable firms to channel more resources into exploitation rather than exploration, thus reducing their capacity to adapt to future environmental changes and new opportunities (Hannan and Freeman, 1984). Davila et al. (2012) recognize the risk of incorporating innovation measurements into a PMS, as they tend to incentivize investment in exploitation innovation activities over exploration innovation activities. Furthermore, Atuahene-Gima (2005) has identified such case of exploitation activities crowding out exploration activities, and Benner and Tushman (2002) have demonstrated that an imbalance in performance measurement is likely to magnify the risk.

Beyond the viewpoint of higher diversity measurement, recent studies have paid more attention to the issue of balance of a PMS. Some scholars believe there is a need for a balance mix of metrics to tailor PMS to the mix of exploitative and exploratory activity (Davila et al., 2012). Furthermore, Bedford (2015) examines the use of MCSs and the effects on firm performance and finds that their combined and balanced use contributes to generating the dynamic tension necessary for managing contradictory innovation modes. Additionally, Bedford et al. (2016) stress that PMSs influence the conversion of intended competence ambidexterity into the achievement of ambidextrous innovation outcomes and PMB plays an important role in the conversion process despite not directly influencing the achievement of innovation outcomes. While extant studies explicitly suggest the importance of balancing performance measurement, we seek to highlight the moderating role of PMB.

Essentially, ambidexterity is associated with paradoxical value manifested in an organization. According to De Long and Fahey (2000), the value is a reflection of the mechanism through which the effect of innovation activity on organizational outcome may be moderated by the culture and management practice internalized in the organization. O’Reilly and Tushman (2008) argue that a kind of culture shaped by top managers can be helpful in resolving the tension between exploiting and exploring. Similarly, Smith and Tushman (2005) point out that top managers excel when they effectively balance strategic contradictions to simultaneously pursue exploitative and exploratory activities. A previous study by Limaj and Bernroider (2017) stresses that management behaviors are moderated by the culture internalized in the organization and that organizational culture can be viewed as the active organism affecting the strength of organizational phenomena. In particular, the internal management practice is one perspective about organizational culture that is in accordance with the essence of culture definition by Schein and Schein (2017).

Furthermore, Given that simultaneous ambidexterity is the organization-level ability, Khan et al. (2017) propose to take into account the role of manager capability in creating the conditions for ambidexterity. Basing on prior studies explaining how managerial capability may alleviate any contradictions between exploitation and exploration, Wu et al. (2019) find that strong managerial capability increases the positive effect of ambidexterity on the innovation performance of Chinese multinational enterprises. In order to analyze the role of organizational design, Vendrell-Herrero et al. (2021) explore management practice of ambidexterity and find that R&D team structure moderates the relationship between IT processes and innovation because it influences the way in which IT is utilized. Generally, the moderating role of some organizational practice is certified to be beneficial of improvement of innovation outcomes.

Drawing explicitly on psychology theory, Bisbe and Malagueño (2012) deal with the use of intervening and moderator variables and examine the effect of strategic PMSs on organizational performance. Monitoring and receiving feedback about the achievement of opposing goals through a balanced PMS will be a source of cognitive conflict among top managers (Bedford et al., 2016). As such, it is important to consider how managers make use of the extensive information available to them and the potential role of psychology theory in management accounting practices (Hall, 2016). It is reasonable to infer that the balance between competing values present in the way of measurement equilibrium would indirectly impact performance due to its effect on individual behaviors. Thus, the extent to which AO impacts IA may be enhanced by the extent to which a balanced PMS is designed for measurement of ambidextrous performance.

In order to facilitate interpretation, we follow the operationalization of PMB by Bedford et al. (2016) and the treatment of absolute difference by Cao et al. (2009), reversing this measurement by subtracting the difference score from 7 so that a higher value indicates greater PMB. The following hypothesis is formalized:

H3: The higher the level of performance measurement balance (PMB) the more positive the association between ambidextrous organization (AO) and innovation ambidexterity (IA).

## Research Method and Design

### Sample Selection and Data Collection

Data for this study were collected using a cross-sectional questionnaire. The target comprised the medium-size companies in high-tech industries—including pharmaceuticals, aerospace vehicles, electronic and communication equipment, computer and office equipment, medical equipment—and other high-tech and medium-tech corporations located in the eastern area of mainland China. We choose corporations located in eastern China as they reflect the future advanced technology.

To collect data, first, we randomly selected the targeted companies listed in a directory of China’s high-tech enterprises by telephone or by sending out an invitation by e-mail to explain the conditions of participation. The questionnaires, in online and spreadsheet forms, were then distributed *via* WeChat operation of PowerSurvey platform (WeChat version of a Chinese DIY survey platform). We also emailed 587 senior- and middle-level managers of 294 companies. After the initial online distribution or mailed surveys to ask the managers for feedback on the questionnaires, follow-ups were conducted *via* telephone calls or emails to company administration office contacts or directly to the managers. The survey was open for 6 months and 152 managers responded. The respondents indicated the extent to which they agreed or disagreed with six statements about how their firm had performed against the three objectives of ambidexterity, regarding organizational strategy over the past 3 years. Valid responses were obtained from 136 senior- and middle-level managers of more than 100 Chinese companies, with a response rate of 23.17%. The surveys were sent to senior- and middle-level managers only, because they have in-depth knowledge on the innovation strategies and operations of their organizations. The participants provided consent to participate in this study. We also got approval from a relevant ethics board. The industry category is based on the definition espoused by the Organization for Economic Co-operation and Development (Hatzichronoglou, 1997) and the descriptive statistics of the sample companies is displayed in Table 1.

**TABLE 1:** Descriptive statistics of the sample companies (*N* = 136).

Sample companies	Category	Frequency	Percent
Employees (unit: person)	<50	15	11.03
	50–300	49	36.03
	301–2000	54	39.71
	>2000	18	13.23
Ownership	Private enterprises	105	77.21
	State-owned enterprises	31	22.79
High-tech	Aerospace	1	0.74
	Computers and office machinery	43	31.63
	Electronics-communications	8	5.88
	Pharmaceuticals	14	10.29
Medium-high-tech	Chemicals	35	25.73
	Other	35	25.73
	Total	136	100

In order to assess possible non-response bias, we first compared the variable means of early respondents with those of late respondents. Next, we compared the industry and size profile of respondents to those of the non-respondents. No significant differences were found in either comparison. Meanwhile, we conducted a Harmann’s single factor test on the survey items used to form the constructs to identify common method bias and found that a single-source bias is not a significant concern.

All items of the questionnaire were originally developed in English, then translated into Chinese and back-translated into English to ensure the accuracy of the meanings, based on the methods of Li and Atuahene-Gima (2001) and Lin et al. (2013). Respondents rated the items on a 7-point Likert-type scale ranging from 1 = strongly disagree to 7 = strongly agree, indicating where they felt their company ranked on each of these items.

### Constructs of Interest and Measurement

Ghoshal and Bartlett (1994) define organizational context in terms of four behavior attributes: discipline, stretch, support, and trust. In order to measure organizational context by developing multi-item scales to represent the dimension of these attributes, Gibson and Birkinshaw (2004) identify two factors to represent the combination of the items developed for them: SS for discipline and stretch, and PM for support and trust. Gibson and Birkinshaw (2004) suggest that when a supportive organizational context enables individuals to engage in both exploitation- and exploration-oriented actions, performance is subsequently improved.

Responding to the emphasis on contextual ambidexterity, we measure SS and PM based on a balanced and comprehensive context. Prior studies examine variations in the design and use of MCSs for different kinds of innovation (McCarthy and Gordon, 2011; Bedford, 2015), arguing that a comprehensive PMS with diversified performance measurement is effective in balancing effort and decisions toward multiple strategies (Dekker et al., 2013). As suggested by Bedford et al. (2016), a balanced representation of the organizational efforts toward exploitation and exploration is important for realizing IA. Furthermore, Hedlund and Ridderstrale (1997) point to the need for a behavioral orientation toward dual capacities, rather than a higher-level separation of those capacities.

Basing our survey items on previous research and on input from an expert panel of academics, we pretested them on a small sample of managers in targeted companies prior to conducting the survey to ensure that meanings were clear. After receiving their feedback, we incorporated as few changes as possible to the final formulations of measurement items. IIS and RIS are based on the version of the instrument developed by Zahra et al. (2000) and used by Atuahene-Gima (2005). Five items are applied for both IIS and RIS, and confirmatory factor analyses show that the four items load as one construct. IPM and RPM are separately assessed through three items derived from the instrument developed by Chiesa et al. (2009) and Bedford et al. (2016). Confirmatory factor analyses show that the three items load as one construct (see Table 2).

**TABLE 2:** Results of confirmatory factor analysis.

Items	IIS	RIS	IPM	RPM	AII	ARI
IIS1	0.872					
IIS2	0.866					
IIS3	0.837					
IIS4	0.867					
RIS1		0.886				
RIS2		0.891				
RIS3		0.907				
RIS4		0.869				
IPM1			0.793			
IPM2			0.846			
IPM3			0.863			
RPM1				0.877		
RPM2				0.720		
RPM3				0.772		
AII1					0.894	
AII2					0.916	
AII3					0.851	
ARI1						0.873
ARI2						0.918
ARI3						0.859
Cronbach’s Alpha	0.88	0.91	0.78	0.78	0.86	0.86
Composite Reliability	0.88	0.90	0.77	0.72	0.87	0.85
Average variance extracted (AVE)	0.66	0.72	0.55	0.51	0.69	0.68

*The formulations of the items are abbreviated. For complete formulations, see Appendix A.*

As indicated by Jansen et al. (2012), IA is a contradictory objective. Following the practice applied by Baía and Ferreira (2019), this study uses IA consisting of achieved incremental innovation (AII) and achieved radical innovation (ARI) to measure ambidextrous performance regarding the selection of innovation outcome variables more closely related to the structure of AO. AII and ARI are also measured using a version of the instrument developed by Atuahene-Gima (2005) and Lin et al. (2013) and used by Bedford et al. (2016). PMB is calculated by subtracting the absolute difference score between the average of the scores of metrics incentivizing incremental innovation and the average of the scores of metrics incentivizing radical innovation from 7. Confirmatory factor analyses indicate that three of the four items related to incremental innovation outcome load on one factor, while three of the four items related to radical innovation outcome load on another factor. All related results of confirmatory factor analyses are shown in Table 2.

### Research Design and Data Analysis

We verify how AO could be embodied as a more comprehensive second-order construct, its impact on IA, and how the PMB moderates the relationship between AO and IA by applying the AMOS program to explain the ambidexterity activity of the organization because the SEM results in a fit index for the whole model rather than partial robustness when applying multiple regression analyses (Kline, 2005). The SEM approach has been widely adopted in many prior studies. Anderson and Gerbing (1988) confirm that the model-building concerning SEM offers great potential for construct validation in the social sciences. In addition, SEM allows for multiple indicators of latent variables and represents a more realistic relationship among the variables under study. It provides a higher-order model with contexts characterized by effectively handling methodological issues such as multicollinearity, uni-dimensionality, and discriminant validity (Agostini et al., 2016).

## Results

### Selection of First-Order Factors and Confirmation of Ambidextrous Organization Structure

In order to identify the most suitable structure of AO, we test five different models of AO constitution, produce the statistical results for the measurement models, and come to some conclusion about the models, including the relationships between AO and first-order factors, reliability and validity test conclusion for first-order constructs, and model fit indicators for all five models, as shown in Table 3. We finally select model one as the most suitable one for further analysis based on acceptable reliability, validity, and model fit index value (Fornell and Larcker, 1981; Hu and Bentler, 1999; Hair et al., 2006; Koufteros and Marcoulides, 2006).

**TABLE 3:** Results of AO integrated as a second-order construct.

	Model 1	Model 2	Model 3	Model 4	Model 5

**Path**	**Path co-efficient (*t*–Value with significance)**				
AO → IIS	0.87 (*t* = 5.44)***	0.89 (*t* = 4.62)***	0.87 (*t* = 4.31)***	0.47 (*t* = 4.79)***	
AO → RIS	0.97 (*t* = 5.29)***	0.94 (*t* = 3.39)***	0.93 (*t* = 7.45)***		0.53 (*t* = 3.82)***
AO → IPM		0.39 (*t* = 3.57)***	0.46 (*t* = 3.84)***	0.73 (*t* = 4.80)***	0.72 (*t* = 5.39)***
AO → RPM	0.68 (*t* = 5.44)***		0.73 (*t* = 5.71)***	1.24 (*t* = 3.36)***	1.27 (*t* = 3.82)***
Verdict on discriminant validity	Supported	No supported	No supported	No supported	No supported
**Model fit index**					
Chi-square	94.95	106.25	232.67	88.49	96.91
CMIN/DF	2.32	2.59	3.19	2.79	3.03
NFI	0.90	0.89	0.82	0.87	0.88
IFI	0.94	0.93	0.87	0.91	0.91
CFI	0.94	0.93	0.86	0.90	0.91
RMSEA	0.079	0.11	0.13	0.12	0.12
Verdict on model fit	Supported	No supported	No supported	No supported	No supported

***Significant at the 0.001 level (Two-tailed test).

*Verdict on discriminant validity is supported when AVE is more than 0.5.*

### Individual Measurement Models and Preliminary Examination

**FIGURE 1:**
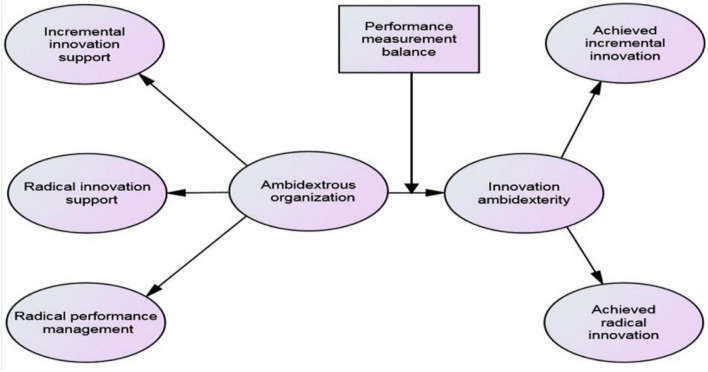
Theoretical higher-order structural model including moderating effect.

We follow the hierarchical approach of Koufteros et al. (2009) to investigate which measurement model best fits the data of our sample group, while acknowledging the multidimensional nature of the higher-order factor. Figure 1 depicts the research model in this paper.

The results shown in Tables 2, 3 indicate sufficient construct reliability and convergent validity according to relevant criteria mentioned by Nunally (1978). Discriminant validities related to Model 2 to Model 5 could not be supported based on the criteria of Fornell and Larcker (1981) because there is no evidence of discriminant validity for every comparison when the squared correlation between any two constructs are compared against their individual average variance extracted (AVE). Given the correlations among the independent variables (IIS, RIS, and RPM) and that between AII and ARI, a multicollinearity issue also seems to emerge. However, issues of discriminant validity and multicollinearity can be handled effectively, and the idiosyncrasy of each facet can be retained with a conceptualization and specification of the higher-order measurement and structural model (Koufteros et al., 2009).

### Hierarchical Approach

A hierarchical approach is usually applied as a systematic process for evaluating alternative models and describing relationships between observed and latent variables (Koufteros et al., 2009). This process includes the construction of different models and the selection of a measurement model. In order to test the second-order model, we follow the approach suggested by Lai (2006) and Koufteros et al. (2009) to construct four different structural models.

Model 1 is hypothesized to include one first-order latent variable with 11 observed indicators. Model 2 contains three first-order uncorrelated factors. Model 3 estimates the correlation factors among the three first-order constructs. Model 4 comprises one second-order factor and three first-order factors with corresponding observed variables; it shows first- and second-order indicators as being reflective and hypothesizes that the second-order construct accounts for the covariance among the three first-order constructs.

The four models are initially compared based on their fit indices; however, selection of the measurement model depends on reliability beyond a mere comparison of fit indices, which means that the selected measurement model is not necessarily the leading model with the best fit indices. Their fit indices are shown in Table 4. Compared to Models 1 and 2, the fit indices of Models 3 and 4 are better, which implies that they fit the data well and are more reliable. According to a study by Arnau and Thompson (2000), although a second-order structural model could not provide a better model fit, this model still performs better than a first-order correlated model when discriminant validity and multicollinearity are considered. Hence, this study supports a second-order model in the context of ambidexterity from an integrated conceptual point of view.

**TABLE 4:** Alternative measure models.

Goodness of fit indices	Model 1: One first-order factor	Model 2: Three first-order factors, uncorrelated	Model 3: Three first-order factors, correlated	Model 4: Three first-order factors, One second-order factor
Chi-square	178.75	253.28	94.95	103.84
CMIN/DF	4.06	5.76	2.32	2.42
NFI	0.82	0.74	0.90	0.90
IFI	0.86	0.78	0.94	0.94
CFI	0.86	0.78	0.94	0.94
RMSEA	0.15	0.19	0.099	0.10

**FIGURE 2:**
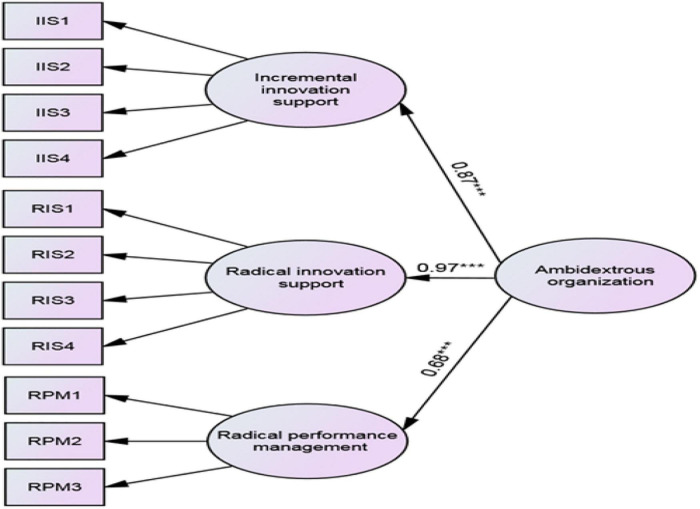
Model 4: Three first-order factors, one second-order factor. *** Significant at the 0.001 level (Two-tailed test).

Finally, according to the above analysis, Model 4 appears to be the most reliable option, with all standardized path coefficients connecting the second-order construct to the first-order variables being statistically significant (see Figure 2). As suggested by Marsh and Hocevar (1985), the target coefficient may be applied as the ratio of the chi-square value of the first-order model to that of the second-order factor model to assess the fit of the second-order factor model relative to the first-order factor model. In this study, a value of 0.91 is less than the upper t-value limit (i.e., 1.0), which means that the second-order factor model could effectively interpret the relationship among the first-order factors.

Following Model 4, the fit indices, along with the *t*-values, provide more evidence of convergent validity associated with first-order factors (see Table 5). Furthermore, the loadings of the first-order factors to the second-order factor and their *t*-values point to a very strong relationship between first-order factors and the second-order factor, attesting to the convergent validity of the representative second-order model. Hence, hypothesis H1 has conditionally been supported (IPM excluded). The reason why IPM is excluded is discussed in section “Discussion.”

**TABLE 5:** Measurement model for three first-order latent factors and one second-order latent factor.

Variable	First-order factor 1: IIS	First-order factor 2: RIS	First-order factor 3:RPM	Second-order factor: OA
IIS1	0.85 (*t* = 11.14)***			
IIS2	0.79 (*t* = 10.12)***			
IIS3	0.78 (*t* = 9.89)***			
IIS4	0.81			
RIS1		0.87 (*t* = 11.81)***		
RIS2		0.86 (*t* = 11.78)***		
RIS3		0.85 (*t* = 11.51)***		
RIS4		0.81		
RPM1			0.79 (*t* = 6.55)***	
RPM2			0.54 (*t* = 5.26)***	
RPM3			0.69	
IIS				0.87 (*t* = 5.44)***
RIS				0.97 (*t* = 5.29)***
RPM				0.68 (*t* = 5.44)***

*** Significant at the 0.001 level (Two-tailed test).

### Model Testing: Analysis of the Structural Model

Choosing model 4 (higher-order model) as the best measurement model, we proceeded to test of the structural model based on this model. We still assumed a structural model where IA is specified as the dependent variable, AO hypothesized as the second-order factor affecting it, and PMB identified as the moderating role (see Figure 1). As anticipated, we also controlled for firm size, ownership, and organizational slack in order to be consistent with previous studies (Atuahene-Gima, 2005; Xie et al., 2021).

Structural equation model is suitable for exploring complex relationship between variables, especially the relationship between latent variables. It can not only reveal the rationality of single path but also the feasibility of the whole structural model. After mean centering the first-order variables and PMB, we create the interaction term in order to run the SEM in Figure 1. First, derived models are presented by entering different variables into the SEM. Derived Model 1 is the base model, only with one independent variable AO, as well as control variables and IA included in the equation. Derived model 2 includes AO and a moderator variable. Model 3 includes AO, a moderator variable, and an interaction term, as well as control variables and IA in the equation. In brief, we take AO, PMB, and interaction term (also control firm size, ownership, and organizational slack) as independent variables, and take IA as the dependent variable to construct regression model. The significance of relationship between interaction term and IA is considered when analyzing a moderating effect. The statistical results of three models are presented in Table 6. The results of the derived models 1, 2, and 3 reveal that an AO has a positive significant effect on IA. Thus, H2 is supported. Moreover, the results in derived Model 3 show that the interaction term “AO × PMB” has a positive effect on innovation ambidexterity. Thus, H3 is absolutely supported.

**TABLE 6:** Results of the structural models.

	Dependent variable:	Innovation ambidexterity (IA)	
Variables	Model 1	Model 2	Model 3
**Independent variable**			
Ambidextrous Organization (AO)	0.61 (*t* = 4.69)***	0.63 (*t* = 4.64)***	0.61 (*t* = 4.63)***
**Moderator variable**			
Performance Measurement Balance (PMB)		−0.09 (*t* = −1.32)	−0.03 (*t* = −0.34)
**Interaction term**			
AO × PMB			0.17 (*t* = 2.03)*
**Control variable**			
Firm size	0.02 (*t* = 0.27)	0.01 (*t* = 0.11)	0.03 (*t* = 0.35)
Firm ownership	−0.14 (*t* = −1.86)	−0.13 (*t* = −1.72)	−0.12 (*t* = −1.65)
Organizational slack	0.06 (*t* = 0.89)	0.07 (*t* = 0.99)	0.11 (*t* = 1.461)

*Models 1, 2, and 3 are saturated models, which means common fit indices are not necessary.*

*Significant at the 0.05 level; *** Significant at the 0.001 level (Two-tailed test).

**FIGURE 3:**
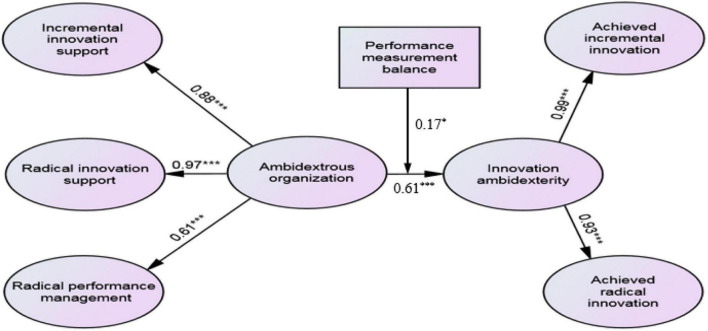
Structural model with significant hypothesis relationships represented. *** Significant at the 0.001 level,* Significant at the 0.05 level (Two-tailed test).

To further interpret the moderation effect, we created one dichotomous variable to classify the organizations into high-balanced and low-balanced PMB groups with a sample size of 68 data sets each. In order to determine whether the difference in the path coefficients is significant, we established the SEM and applied a pairwise comparison test, following the process used by Chin (2000). The multi-group moderation test statistics show that the path coefficient from AO to IA for high-balanced PMB is significantly stronger than the corresponding path in the model for the low-balanced PMB [model comparison index: minimum chi-square (CMIN) = 4.08, *P* = 0.043]. Figure 3 displays the hypotheses and associations supported by our findings.

## Discussion

Focusing on innovation management and performance improvement of AO, the aim of this study is to investigate whether an AO could be conceptualized in a unique, integrated way as a second-order construct encompassing specifically balanced contextual and PM approaches in order to manage the tension between exploitation and exploration. It tries to explain the innovation management of AO by providing a conceptual advance that connects a diversified and balanced context of lower-level constructs to a higher-level construct and IA in an integrated way.

Our findings reveal several aspects relevant to the debate on AO and IA. First, drawing on prior conceptual and empirical research on AO (Chang and Hughes, 2012; Agostini et al., 2016), this study proposes an integrated conceptual model of AO and SEM. It treats the integration of IIS, RIS, and RPM—rather than that of structural design and contextual design indicated in previous studies (Nosella et al., 2012; O’Reilly and Tushman, 2013; Agostini et al., 2016)—as the essence of AO in resolving strategic dilemmas. The results reveal that this model is better than other potential competing models and empirically validate the claim that integrating emphasizes the interdependencies between contradictory strategic objectives (Bedford et al., 2016).

Second, our study indicates that the second-order theoretical model fits the data well in a unique, integrated way and AO, represented by a balanced higher-order construct, positively affects IA outcomes. It has verified that, instead of structural ambidexterity, balanced competence ambidexterity and RPM can be effectively applied as factors of the second-order construct. It emphasizes that, in view of the nature of ambidextrous innovation and the tension between exploitation and exploration, the design of balanced dimensions of SS and PM is helpful in resolving strategic dilemmas and in pushing two different kinds of AO innovation simultaneously.

Third, our testing of the relationship between AO and IA indicates that RPM, rather than IPM, may be integrated into a second-order construct. Contrary to the long-held viewpoint of exploitation crowding out exploration efforts, we find the risk of exploration crowding out exploitation efforts in Chinese high-tech firms. This implies that Chinese managers intend to motivate employee behavior by emphasizing exploratory activity and radical innovation when considering ambidextrous innovation and maintaining balance between exploitative and exploratory activities, which supports the conclusion that technology latecomers are more inclined to carry out near-radical innovation (Chen et al., 2020a). Meanwhile, SS plays a more important role than PM in integration of OA.

Lastly, balance in performance measurement is critical for Chinese high-tech firms pursuing IA because simultaneous ambidexterity is considered necessary for enterprises’ survival and future development. This study demonstrates that although PMB does not directly impact IA, it plays a moderating role in improving IA, exceeding the previously studied buffers of a significant role in the management of innovation (Chenhall and Moers, 2015), multiple strategic priorities (Dekker et al., 2013), and incentive to incremental and radical innovation (Bedford et al., 2016).

## Implications

The results of this study provides unique insight into the management of AO and IA. They also provide implications for the innovation management of firms, especially technology latecomers, in a non-Western and emerging economy context.

### Theoretical Implications

Ours study makes several important contributions to the existing literature referring to organizational ambidexterity. Firstly, it challenges the existing wisdom. Contrary to the long-held viewpoint of exploitation crowding out exploration efforts, we come to a different conclusion and find the risk of exploration crowding out exploitation efforts in Chinese high-tech firms.

Secondly, the results from this study extends our understanding of the integration of three organizational solutions and balanced performance measurement into a higher-order construct representing AO and IA. Thus far, there have been scarce studies that have examined the comprehensive effect of management practice on balanced innovation outcomes through an integrated SEM based on configurational theory. In this study, higher-order modeling is an important step forward regarding dealing with ambidexterity, as suggested by Gibson and Birkinshaw (2004); Kauppila (2010), and Chang and Hughes (2012). Our study, being different from previous research by McCarthy and Gordon (2011) and Agostini et al. (2016), focuses on a configurational approach and points to both balanced contextual and PM elements as keys to the sophisticated process needed to achieve both incremental and radical innovation outcomes. It shows that the integration of IIS, RIS, and specific RPM, rather than the integration of structural design and contextual design indicated in previous studies (Nosella et al., 2012; O’Reilly and Tushman, 2013; Agostini et al., 2016), may be a better model of AO for technology latecomers compared to technology incumbents in resolving strategic dilemmas.

Thirdly, our findings also reveal that PMB significantly moderates the relationship between AO and IA and plays an important role in the process of improving IA. On the one hand, RPM, rather than IPM, may be integrated into the second-order construct, which indicates that despite the assumed uncertainty of exploration benefit enabling firms to assign more resources into exploitation rather than exploration in previous literature (Benner and Tushman, 2002; Atuahene-Gima, 2005; Bedford et al., 2016), it is verified that Chinese high-tech firms are inclined to carry out near-radical innovation rather than incremental innovation. On the other hand, the moderating role of PMB means that it is necessary for technology latecomers to cultivate an innovation culture pursuing exploitation and exploration simultaneously and encouraging behaviors of individuals toward ambidextrous innovation. PMB may be beneficial in creating a company culture where managers support the balanced development of competence ambidexterity (Leonard-Barton, 1992; Levinthal and March, 1993) and manage reversed contradictory demands (i.e., exploration activities crowding out exploitation activities) through an integrated mechanism and a balanced PMS.

### Practical Implications

The implications of this study for business practice are also important. The findings support the idea that balanced and diversified SS may be more necessary than PM for managers to create an AO. Meanwhile, the balanced solution of ambidextrous performance measurement makes it possible for entrepreneurs and managers to manage the performance of organizations from the perspective of ambidextrous innovation outcomes. Our findings provide real significance for performance management of Chinese high-tech firms, and also emphasize practical contributions for organizations of other emerging countries in the Asia Pacific, which means it is possible for high-tech firms to effectively manage IA.

This study also demonstrates that the relevance of PMS for innovation is not solely a form of presentation in an integrated way, but also highly dependent on how managers apply it to motivate employee behavior to improve IA, which requires the use of some type of mechanism (Lawson et al., 2009). Our findings reveal that it is necessary for high-tech firms to pay more attention to the balance of PM, especially for technology latecomers if they need to pursue ambidextrous innovation and improve IA.

## Limitations and Future Research

Our attempt to develop the theory of ambidextrous innovation management of organizations has some limitations. First, we have investigated the impact of an AO on IA, appreciated higher-order modeling, and found that a second-order construct could be used to resolve dilemma. However, whether the structure may be verified in a larger sample size through a higher-order construct remains unknown. Second, given the cross sectional nature of individuals in sample, it is usually not possible to strictly draw causal relationships, which means that relevant results represent usually necessary but not sufficient conditions for causality. Meanwhile, a cross-sectional analysis does not capture the dynamic nature of organization. Third, we should incorporate more control variables, such as firm age and industry, into research model. They perhaps impact research results significantly. Future research should include variables such as firm age/experience, years of professional experience, and other business environment variables such as cultural and dynamic environment in the analysis. In this study, PMB plays a positive role in moderating the relationship between OA and IA. However, the type of organizational culture that is helpful for the form of PMB still remains unexplored. Therefore, future studies should further explore how to form unique organizational culture and to integrate management accounting practices to improve organizational innovation outcomes.

## Conclusion

In order to fully appreciate higher-order modeling incorporating AO and IA, we sought to explain innovation management of the organization by providing a conceptual advance that relates a diversified and balanced solution of the context of lower-level constructs to higher-level constructs and a balanced measurement of IA in an integrated way. To achieve the purpose, we compared different measurement models to identify the most relevant one that best fits the data.

Our results reveal that the second-order theoretical model fits the data well and the contextual approach and RPM as the components of a higher-order construct of AO help improve IA in a unique, integrated way. Differing from the existing research on three underlying dimensions that define the structure and context needed to pursue IA, this study presents a unique integration of a higher-order construct and attempts to extend academic ideas of innovation management related to AO. We also find the risk of exploration crowding out exploitation efforts in Chinese high-tech firms. Additionally, one finding of the second-order model indicates that RPM is an indispensable first-order factor of OA associated positively with the outcome of ambidextrous innovation, which implies that Chinese managers may intend to motivate employee behavior by emphasizing exploratory activity and radical innovation when considering ambidextrous innovation and maintaining a balance between exploitative and exploratory activities, which is consistent with the conclusion that technology latecomers are more inclined to carry out near-radical innovation (Chen et al., 2020a).

Another result of our study reveals that PMB plays a moderating role in improving IA although it does not directly affect IA, which means that it is necessary for high-tech firms to apply not only a PMS in pursuing ambidextrous innovation, but also a balanced PM design in PM to improve IA.

## Data Availability Statement

The raw data supporting the conclusions of this article will be made available by the authors, without undue reservation.

## Ethics Statement

The studies involving human participants were reviewed and approved by the Ethics Committee of Jiangsu University of Technology. The Ethics Committee waived the requirement of written informed consent for participation.

## Author Contributions

RC substantially contributed to the design of the work, collected the data for the study, prepared the draft of the manuscript and reviewed it critically, conducted the qualitative and quantitative analyses, and provided all tables. RJ modified some pages’ contents of the draft and provided all figures. Both authors contributed to the article and approved the submitted version.

## Conflict of Interest

The authors declare that the research was conducted in the absence of any commercial or financial relationships that could be construed as a potential conflict of interest.

## Publisher’s Note

All claims expressed in this article are solely those of the authors and do not necessarily represent those of their affiliated organizations, or those of the publisher, the editors and the reviewers. Any product that may be evaluated in this article, or claim that may be made by its manufacturer, is not guaranteed or endorsed by the publisher.

## Appendix A

**Table:**

Latent variable: Incremental innovation support (IIS). Over the past three years,
IIS1: Your company has been investing in enhancing capabilities in exploiting mature technologies of the industry that improve efficiency of current product/service innovation practices.
IIS2: Your company has been enhancing capabilities in seeking for solutions to customer problems that are near to existing solutions.
IIS3: Your company has been upgrading capabilities in product/service research and development processes in which your company already have gained experience.
IIS4: Your company has been strengthening knowledge and skills for projects that improve efficiency of existing product/service innovation activities.
Latent variable: Radical innovation support (RIS). Over the past three years,
RIS1: Your company has been learning product/service development skills and processes entirely new to your industry.
RIS2: Your company has been acquiring product/service technologies and skills entirely new to your company.
RIS3: Your company has been developing new skills in the relevant areas for key product/service innovation.
RIS4: Your company has been strengthening product/service innovation skills in areas where it had no prior experience.
Latent variable: Incremental performance management (IPM). Over the past three years,
IPM1: Your company has applied number of improved products/services used as a performance measurement indictor.
IPM2: Your company has applied profit ratio of improved products/services to total used as a performance measurement indictor.
IPM3: Your company has applied ROI of improved products/services used as a performance measurement indictor.
Latent variable: Radical performance management (RPM). Over the past three years,
RPM1: Financial resources specifically devoted to more radical type innovation projects were regarded as your company’s performance measurement index.
RPM2: Number of new patents for more projects granted each year was regarded as your company’s performance measurement index.
RPM3: Portfolio of products/services was analyzed by breakeven time of incremental innovation projects.
Latent variable: Achieved incremental innovation (AII). Over the past three years,
AII1: Your company has frequently introduced improved products/services into markets.
AII2: Your company has introduced more improved products/services than major competitors.
AII3. Your company has sold more percentage of total sales from new incremental product/service innovations than major competitors.
Latent variable: Achieved radical innovation (ARI). Over the past three years,
ARI1: Your company has frequently launched brand new products/services into markets.
ARI2: Your company has introduced more brand-new products/services than major competitors.
ARI3: Your company has sold more percentage of total sales from new radical product/service innovations than major competitors.
